# Supplementary material for: Large Language Models in Action: Supporting Clinical Evaluation in an Infectious Disease Unit
Source: Healthcare (Basel). 2025 Apr 11;13(8):879. doi: 10.3390/healthcare13080879 (PMC12027404; doi:10.3390/healthcare13080879)
Supplement: Supplementary file 1 [file healthcare-13-00879-s001.zip › healthcare-3504559-supplementary.pdf]

Supplementary Material

Prompts provided to ChatGPT

Antibiotic therapy appropriateness

You are tasked with analyzing patient medical records written in Italian and cross-referencing them with antibiotic therapy guidelines (provided separately) to assess the appropriateness of the administered therapies. Follow these steps:

1. **Identify therapies**: Extract all antibiotic therapies documented in the patient's records, including:
    - The name of the antibiotic.
    - Relevant clinical context or condition (e.g., infection type, underlying conditions).
2. **Evaluate appropriateness**: For each antibiotic therapy:
    - Compare it against the provided guidelines.
    - Determine if the therapy is **appropriate** or **inappropriate** based on the patient's condition and clinical context.
    - Provide a brief justification, referencing the relevant guideline recommendations. If a therapy is inappropriate, suggest alternatives.
3. **Structure your response**: Clearly separate:
    - The list of identified therapies.
    - The appropriateness evaluation and justification for each therapy.

Patient Analysis

- **Administered Antibiotic Therapies**:
    - **Therapy 1**: [Antibiotic name, context/condition].
    - **Therapy 2**: [Antibiotic name, context/condition].
- **Appropriateness Evaluation**:
    - **Therapy 1**: [Appropriate/Inappropriate].
        - Justification: [Explain based on the guidelines].
    - **Therapy 2**: [Appropriate/Inappropriate].
        - Justification: [Explain based on the guidelines].

Use concise and professional language. Focus on clinical accuracy and avoid unnecessary details unless they impact the evaluation.

Isolation Measures appropriateness

You are tasked with analyzing patient medical records written in Italian and cross-referencing them with isolation measure guidelines (provided separately) to assess whether the chosen measures were appropriate for the type of infection. Follow these steps:

1. **Identify isolation measures**: Extract all documented isolation measures applied to the patient, including:
   - The type of isolation (e.g., contact, airborne, droplet).
   - The clinical context or type of infection for which the measures were applied.
2. **Evaluate appropriateness**: For each isolation measure:
   - Compare it against the provided guidelines.
   - Determine if the measure is **appropriate** or **inappropriate** based on the patient's infection type and clinical context.
   - Provide a brief justification, referencing the relevant guideline recommendations. If the measures are inappropriate, suggest corrections.
3. **Structure your response**: Clearly separate:
   - The identified isolation measures.
   - The appropriateness evaluation and justification for each measure.

Patient Analysis

- **Isolation Measures Applied**:
  - **Measure 1**: [Type of isolation, context/infection type].
  - **Measure 2**: [Type of isolation, context/infection type].
- **Appropriateness Evaluation**:
  - **Measure 1**: [Appropriate/Inappropriate].
    - Justification: [Explain based on the guidelines].
  - **Measure 2**: [Appropriate/Inappropriate].
    - Justification: [Explain based on the guidelines].

Use concise and professional language. Focus on clinical accuracy and avoid unnecessary details unless they impact the evaluation.

Ulcer Management appropriateness

You are tasked with analyzing patient medical records (file: "Paziente 1.pdf") written in Italian and cross-referencing them with pressure ulcer management guidelines (provided separately) to assess whether the wound care was appropriate (if ulcer pressure was detected). Specifically, evaluate the following:

1. Was the chosen dressing appropriate for the stage of the pressure ulcer?

2. Was the dressing changed at the recommended frequency according to the guidelines for that stage?

Follow these steps:

1. **Identify pressure ulcer management details**: Extract all documented information related to pressure ulcer care, including:
   - The stage of the ulcer.
   - The type of dressing used.
   - The frequency of dressing changes (if specified).
2. **Evaluate appropriateness**: For each documented ulcer:
   - Compare the dressing choice against the guidelines to determine if it was **appropriate** or **inappropriate** for the ulcer stage.
   - Assess the frequency of dressing changes against the guidelines and determine if it was **appropriate** or **inappropriate**.
   - Provide a brief justification for both evaluations, referencing relevant guideline recommendations.
3. **Structure your response**: Clearly separate:
   - The pressure ulcer management details.
   - The appropriateness evaluation for dressing choice and frequency of changes.

Patient Analysis

- **Pressure Ulcer Management**:
  - **Ulcer 1**: [Stage, dressing type, frequency of changes].
  - **Ulcer 2**: [Stage, dressing type, frequency of changes].
- **Appropriateness Evaluation**:
  - **Ulcer 1**:
    - **Dressing Choice**: [Appropriate/Inappropriate].
      - Justification: [Explain based on the guidelines].
    - **Frequency of Changes**: [Appropriate/Inappropriate].
      - Justification: [Explain based on the guidelines].
  - **Ulcer 2**:
    - **Dressing Choice**: [Appropriate/Inappropriate].
      - Justification: [Explain based on the guidelines].
    - **Frequency of Changes**: [Appropriate/Inappropriate].
      - Justification: [Explain based on the guidelines].

Use concise and professional language. Ensure clinical accuracy and avoid unnecessary details unless they impact the evaluation.

Urinary Catheter appropriateness

You are tasked with analyzing patient medical records written in Italian and cross-referencing them with urinary catheter management guidelines (provided separately) to assess whether the catheter care was appropriate. Specifically, evaluate the following:

1. Was the urinary catheter placement performed appropriately based on clinical necessity?
2. Was the catheter removed or replaced according to the recommended timeline in the guidelines?

Follow these steps:

1. **Identify catheter management details**: Extract all documented information related to urinary catheter use, including:
   - The clinical reason for catheter placement.
   - The date of placement and removal/replacement (if specified).
   - Any relevant notes about the patient's clinical condition or catheter-related care.
2. **Evaluate appropriateness**: For each documented catheter:
   - Determine if the placement was **appropriate** or **inappropriate** based on clinical necessity and the provided guidelines.
   - Assess if the catheter was removed or replaced **appropriately** or **inappropriately** according to the recommended timeline in the guidelines.
   - Provide a brief justification for both evaluations, referencing relevant guideline recommendations.
3. **Structure your response**: Clearly separate:
   - The catheter management details.
   - The appropriateness evaluation for placement and removal/replacement.

Patient Analysis

- **Urinary Catheter Management**:
  - **Catheter 1**: [Reason for placement, placement date, removal/replacement date].
  - **Catheter 2**: [Reason for placement, placement date, removal/replacement date].
- **Appropriateness Evaluation**:
  - **Catheter 1**:
    - **Placement**: [Appropriate/Inappropriate].
      - Justification: [Explain based on the guidelines].
    - **Removal/Replacement**: [Appropriate/Inappropriate].
      - Justification: [Explain based on the guidelines].
  - **Catheter 2**:
    - **Placement**: [Appropriate/Inappropriate].
      - Justification: [Explain based on the guidelines].
    - **Removal/Replacement**: [Appropriate/Inappropriate].
      - Justification: [Explain based on the guidelines].

Use concise and professional language. Ensure clinical accuracy and avoid unnecessary details unless they impact the evaluation.

Infusion Line Management appropriateness

You are tasked with analyzing patient medical records written in Italian (file: "Paziente 1.pdf") and cross-referencing them with infusion line management guidelines (provided separately) to assess whether the management of infusion lines was appropriate. Specifically, evaluate the following:

1. Was the placement of the infusion line appropriate based on clinical necessity?
2. Were the maintenance practices (e.g., flushing, dressing changes) and removal performed according to the recommended guidelines?

Steps**:**

1. **Identify infusion line details**: Extract all documented information related to infusion line use, including:
   - The type of line (e.g., CVC, PICC, Midline, peripheral venous catheter).
   - The clinical reason for placement.
   - The placement date.
   - Maintenance details (e.g., flushing protocols, dressing changes).
   - Removal or replacement date (if specified).
2. **Evaluate appropriateness**: For each type of infusion line:
   - Determine if the placement was **appropriate** or **inappropriate** based on clinical necessity and the guidelines.
   - Assess if maintenance and removal/replacement were performed **appropriately** or **inappropriately** based on the guidelines.
   - Provide a brief justification for both evaluations, referencing relevant guideline recommendations.
3. **Structure your response**: Clearly separate:
   - The infusion line management details by type of line.
   - The appropriateness evaluation for placement, maintenance, and removal/replacement.

Patient Analysis

- **Infusion Line Management**:
  - **Line Type**: [e.g., CVC, PICC, Midline, etc.].
    - **Reason for Placement**: [Clinical context].
    - **Placement Date**: [If available].
    - **Maintenance Details**: [Flushing, dressing changes, etc.].
    - **Removal/Replacement Date**: [If available].
- **Appropriateness Evaluation**:
  - **Placement**: [Appropriate/Inappropriate].
    - Justification: [Explain based on the guidelines].
  - **Maintenance**: [Appropriate/Inappropriate].

    - Justification: [Explain based on the guidelines].
  - **Removal/Replacement**: [Appropriate/Inappropriate].
    - Justification: [Explain based on the guidelines].

Notes:

- For patients with multiple lines, list them separately under their respective types.
- If maintenance details are complex, summarize the key aspects and reference relevant sections in the patient records or guidelines.
